# Patient selection for PRRT rechallenge in neuroendocrine tumors: impact of initial response duration on outcomes

**DOI:** 10.1007/s00259-026-08130-8

**Published:** 2026-08-14

**Authors:** Philipp E. Hartrampf, Aleksander Kosmala, Sophie Alwers, Franziska Lutz, Sebastian E. Serfling, Natalie Hasenauer, Christof Däubler, Alexander Meining, Andreas K. Buck, Rudolf A. Werner, Alexander Weich

**Affiliations:** 1 Department of Nuclear Medicine, University Hospital Wuerzburg, Oberdürrbacherstraße 6, 97080 Wuerzburg, Germany; 2 University Hospital Wuerzburg, ENETS Center of Excellence, Wuerzburg, Germany; 3 Department of Internal Medicine II, Gastroenterology, University Hospital Wuerzburg, Wuerzburg, Germany; 4 Department of Nuclear Medicine, University Hospital, Ludwig-Maximilians-Universitaet Muenchen, Munich, Germany

**Keywords:** DOTATOC, DOTATATE, GEP NET, PRRT, PFS, Retreatment

## Abstract

**Background:**

Patient selection for rechallenge peptide receptor radionuclide therapy (re-PRRT) in neuroendocrine tumors (NET) remains poorly defined. As re-PRRT is increasingly considered in clinical practice, identifying patients most likely to benefit has become a critical yet unresolved question. This study evaluated the efficacy and safety of re-PRRT and aimed to identify pragmatic factors associated with retreatment benefit.

**Materials and methods:**

This retrospective study included 37 patients with neuroendocrine tumors (NET). Kaplan–Meier estimates were used to evaluate progression-free survival (PFS) following initial (i-PRRT) and re-PRRT. Baseline laboratory parameters were compared between treatment courses, and SSTR-targeted PET metrics (SUV_mean_, SUV_max_, and SSTR-positive tumor volume) were assessed at baseline and follow-up. Univariable Cox regression identified prognostic factors for PFS. Adverse events were graded according to CTCAE v5.0.

**Results:**

Median PFS was 32 months after i-PRRT and 17 months after re-PRRT, with disease progression observed in 20/37 patients. Longer PFS following initial therapy was associated with improved outcome after retreatment (per month, HR 0.96, 95% CI 0.93–0.999; *p* < 0.05). In an exploratory analysis, patients with an initial PFS exceeding 24 months demonstrated a longer median PFS after re-PRRT than those with shorter initial PFS (19 vs. 4 months; log-rank *p* = 0.02). Higher baseline CRP was significantly associated with shorter PFS after i-PRRT (per mg/dl, HR 8.8, 95% CI 1.2–58.6; *p* < 0.05), whereas no such association was observed for re-PRRT (per mg/dl, HR 0.79, 95% CI 0.13–3.8; *p* = 0.8). Instead, higher baseline LDH was significantly associated with shorter PFS following re-PRRT (per U/l, HR 1.007, 95% CI 1.000–1.014; *p* < 0.05). No PET-derived baseline parameter correlated with PFS. RECIST indicated predominantly stable disease across treatment courses, although responses were less favorable at retreatment. No grade ≥ 3 toxicities were observed during re-PRRT.

**Conclusion:**

Re-PRRT represents a feasible treatment option for selected NET patients. Duration of disease control following initial PRRT was associated with outcome after re-PRRT and may represent a clinically relevant marker for patient selection. The exploratory 24-month threshold requires prospective validation before clinical implementation.

**Supplementary Information:**

The online version contains supplementary material available at https://doi.org/10.1007/s00259-026-08130-8.

## Introduction

 Neuroendocrine neoplasms (NEN) constitute a heterogeneous group of neoplasms that account for approximately 2% of all malignancies [1, 2]. Although NEN can develop in any epithelialized organ, the majority of cases (57.8%) are observed in the gastrointestinal tract, where they are classified as gastroenteropancreatic neuroendocrine neoplasms (GEP-NEN) [3, 4].

Most NEN are differentiated neuroendocrine tumors with a good prognosis, although 20–50% of patients present with distant metastases at diagnosis [3–6]. Standard systemic treatment consists of the administration of cold somatostatin analogues (SSA) [7]. Additional treatment options for patients with advanced neuroendocrine tumors (NETs) include locoregional therapies, such as radioembolization, and systemic therapies, which include interferon-α, sunitinib, or everolimus. The use of systemic chemotherapy is recommended in advanced pancreatic-NETs and in NEN G3 [7]. Peptide receptor radionuclide therapy (PRRT) has emerged as an effective treatment option for patients with progressive disease during SSA therapy [8].

PRRT significantly improved progression-free survival and quality of life in the NETTER-1 trial compared with high-dose octreotide [9–11]. During the long-term follow-up period, only three patients (3%) in the PRRT group experienced treatment-related serious adverse events of grade 3 or higher [10]. In light of these findings, Lutathera^®^ ([^177^Lu]Lu-DOTATATE) has been granted approval as a radiopharmaceutical for the treatment of non-resectable or metastatic, progressive, well-differentiated (G1 and G2), SSTR-positive GEP-NET [12, 13]. Recent randomized trials such as NETTER-2 and COMPETE further underscore the expanding role of PRRT across disease stages [14, 15].

Despite these advances, most advanced NET remain incurable, and disease progression after PRRT is frequently observed. Subsequent treatment options are limited and often associated with greater toxicity, including targeted therapies and cytotoxic chemotherapy [16–18]. This therapeutic gap has generated increasing interest in PRRT retreatment. Retrospective studies suggest that rechallenge PRRT (re-PRRT) may provide clinical benefit, albeit with reduced efficacy compared with initial therapy (i-PRRT), while maintaining a favorable safety profile [19–21]. The present study aimed to expand real-world evidence on re-PRRT in progressive NET and to identify clinically applicable factors associated with outcomes and to explore whether the duration of disease control following initial PRRT can serve as a pragmatic threshold to guide retreatment decisions.

## Materials and methods

### Patient cohort

**Fig. 1:**
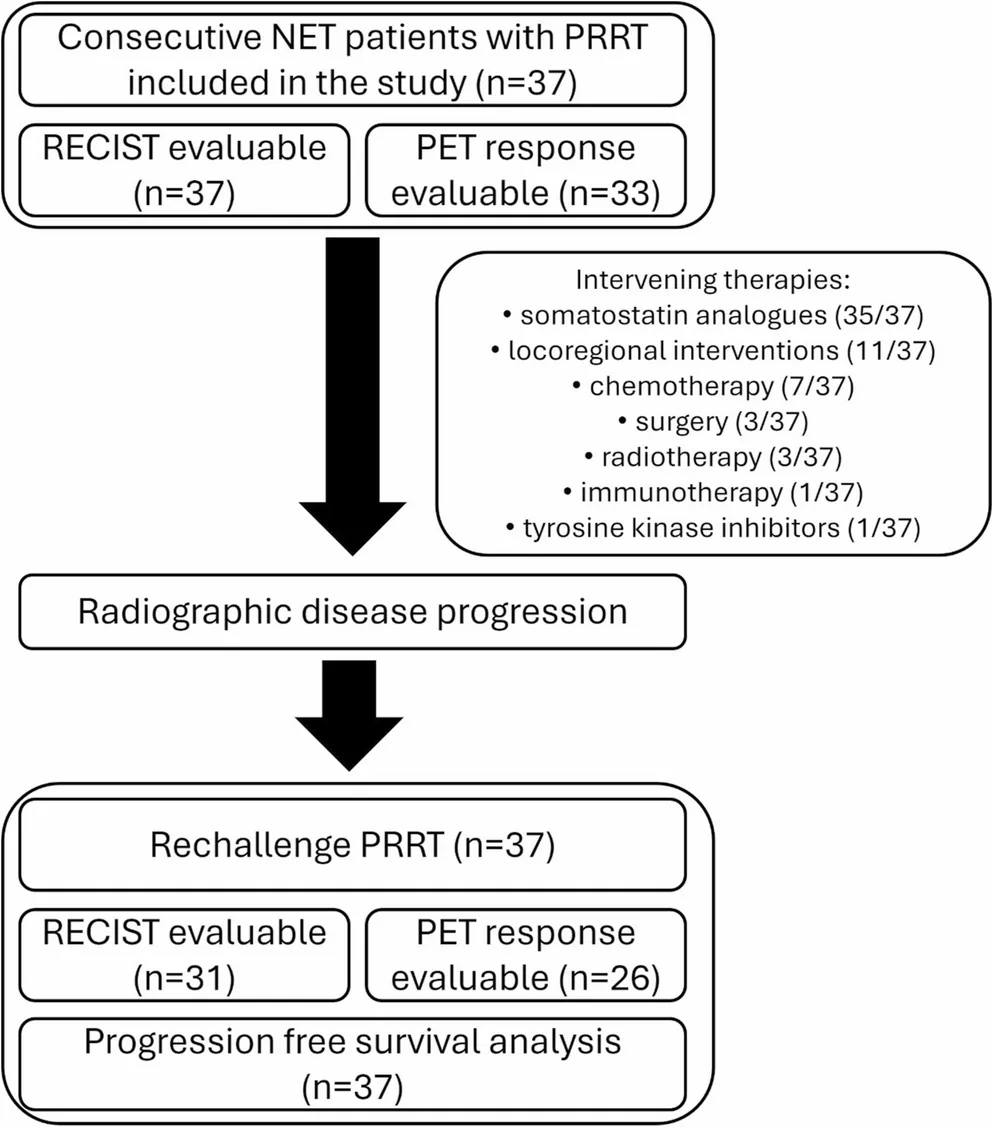
STROBE-style patient flow diagram. Consecutive NET patients included in the study (*n* = 37) underwent both initial PRRT (i-PRRT) and rechallenge PRRT (re-PRRT). The diagram illustrates the availability of RECIST and PET response assessments, the intervening therapies administered between treatment courses, and the datasets available for progression-free survival analyses

This retrospective single-center study included all consecutive patients with histologically confirmed NET who underwent both i-PRRT and re-PRRT, irrespective of tumor grade, primary site, or functional status at the University Hospital of Würzburg (Fig. 1). Eligible patients received [^68^Ga]Ga-DOTATOC PET/CT prior to the initiation of radioligand therapy and after every two treatment cycles. Survival data were collected for all patients. The study was approved by the local ethics committee, which waived the requirement for additional informed consent due to the retrospective study design (waiver number 20231220 01).

### Therapy work-up

Patients underwent whole-body [⁶⁸Ga]Ga-DOTATOC PET/CT before treatment initiation and 8–12 weeks after the second PRRT cycle, with CT performed for attenuation correction and anatomical co-registration. When clinically indicated, a diagnostic contrast-enhanced CT was acquired as part of the PET/CT examination, thereby obviating the need for an additional standalone CT scan. Details regarding radiotracer synthesis and imaging protocols are provided in the Supplementary Material. Blood samples were obtained at each treatment cycle to assess renal and hepatic function, inflammatory markers (C-reactive protein (CRP)), tumor activity (lactate dehydrogenase (LDH)) and hematologic parameters. Patient histories were retrospectively reviewed from electronic medical records.

### Image analysis

Treatment response was assessed according to RECIST 1.1 criteria [22]. RECIST response was assessed based on follow-up imaging at predefined timepoints after two cycles of therapy, when available. PET/CT images were analyzed using Syngo.via software (version VB60, Siemens Healthineers, Erlangen, Germany) for visual interpretation and the Beth Israel plug-in for FIJI (ImageJ) for semi-automated lesion segmentation [23]. Standardized uptake values (SUV_max_, SUV_peak_, and SUV_mean_) across all lesions and total somatostatin receptor–avid tumor volume (SSTR-TV) were calculated using a fixed SUV threshold of 3 (primary reader, S.A.). All segmentations were reviewed by an experienced reader (P.H.), with uncertain cases adjudicated by a third reader (R.A.W.). Total lesion SSTR (TL-SSTR) was derived by multiplying SSTR-TV by SUV_mean_. Changes (Δ) between baseline and follow-up were calculated for all quantitative PET parameters for both treatment courses.

In an exploratory PET response analysis, partial response was defined as a reduction in SSTR-TV greater than 30%, while progressive disease was defined as an increase exceeding 30%; intermediate changes were classified as stable disease. These thresholds were selected pragmatically for exploratory analyses and do not represent validated PET response criteria. Restaging imaging performed 8–12 weeks after two and four PRRT cycles is summarized in Supplementary Table 1.

For progression-free survival analyses, all available follow-up imaging was considered, ensuring complete PFS data for the cohort. All patients were followed until radiographic progression or last available follow-up.

### Treatment protocol

[^177^Lu]Lu-DOTATOC was synthesized in-house and administered to 33/37 patients during i-PRRT and 24/37 patients during re-PRRT. The full details of the radiotracer synthesis and imaging procedures are provided in the Supplementary Material. Patients typically received 7.4 GBq per cycle at 12-week intervals, with a median of 4 cycles (range 2–8) for i-PRRT and 3 cycles (range 1–4) for re-PRRT. Lutathera^®^ was administered according to the manufacturer’s instructions in 4/37 patients during i-PRRT and 13/37 patients during re-PRRT. The choice between [^177^Lu]Lu-DOTATOC and [177Lu]Lu-DOTATATE was based on institutional availability with treatment applied within the scope of regulatory approval whenever possible, and comparable administered activities per cycle.

### Statistical analysis

Statistical analyses were performed using GraphPad Prism version 9.3.0 (GraphPad Software, San Diego, CA). Data are reported as medians with ranges. Paired variables were compared using the Wilcoxon matched-pairs test. The frequency of CTCAE-defined adverse events between patients receiving [¹⁷⁷Lu]Lu-DOTATOC and [¹⁷⁷Lu]Lu-DOTATATE was compared using Fisher’s exact test. Progression-free survival following i-PRRT (PFS1) was defined as the time from treatment initiation to radiographic progression according to RECIST 1.1, while PFS after re-PRRT (PFS2) was defined analogously from the start of re-PRRT. The interval between i-PRRT and re-PRRT was defined as the time between the last cycle of initial PRRT and the first cycle of retreatment. Correlation between the change in tumor volume on PET and the change in diameters on CT were assessed using Spearman’s rank correlation coefficient. Associations between baseline variables and PFS were evaluated using Cox regression. All Cox regression analyses were exploratory and univariable. Hazard ratios for laboratory parameters are reported per unit increase according to the respective measurement scale. No adjustment for multiple testing was performed; therefore, these analyses should be interpreted as hypothesis-generating. Survival curves were generated with the Kaplan–Meier method and compared using the log-rank test. Comparison of PFS1 and PFS2 was done descriptively, recognizing that both endpoints were obtained from the same patients. Hazard ratios (HRs) are presented with 95% confidence intervals (CIs), and statistical significance was defined as *p* < 0.05. Given the retrospective nature of the study, no formal sample size calculation was performed, and all eligible patients within the study period were included.

## Results

### Patients’ characteristics

**Fig. 2:**
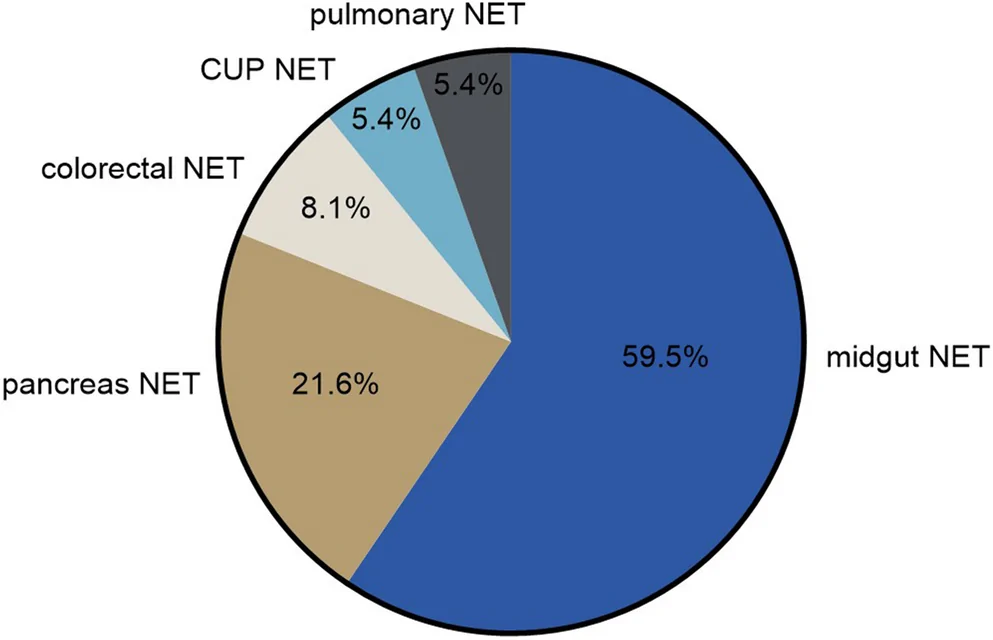
Proportion of the different NET subgroups in the cohort

A total of 37 patients with neuroendocrine tumors (NETs) and a median age of 63 years (range 24–80 years) at the start of i-PRRT were included in the study. The majority of patients (59.5%) suffered from midgut NET, followed by those with pancreatic NET (21.6%) (Fig. 2). The largest cohort consisted of G2 NET patients (76%, Table 1).

**Table 1:** Patients‘ characteristics

Clinical Variables	i-PRRT	re-PRRT	*p*-value
	*N* (range)	*N* (range)	
Age at first cycle of PRRT (years)	63 (24–80)		
male	22 (59%)		
G1 tumor	6 (16%)		
G2 tumor	28 (76%)		
G3 tumor	1 (3%)		
Unknown grading	2 (5%)		
Treatment cycles per patient	4 (2–8)	3 (1–4)	
Median Ki-67 index (range)	5% (1–40%)		
Cumulative activity (GBq)	29.5 (13.3–60.8)	22.2 (5.0-30.4)	
*Baseline laboratory values*			
CRP (mg/dl)	0.25 (0.02–3.08)	0.40 (0.09–9.55)	0.24
LDH (U/l)	202 (143–336)	200 (128–451)	0.93
Hemoglobin (g/dl)	13.2 (10.1–17.2)	12.2 (9.4–16.9)	0.002
Creatinine (mg/dl)	0.89 (0.54–1.57)	0.97 (0.44–1.7)	0.08
eGFR (CKD-EPI) (mL/min/1.73 m²)	85 (44–114)	74 (40–113)	0.001
Bilirubin (mg/dl)	0.4 (0.2–0.9)	0.5 (0.2–1.3)	0.14
AST(U/l)	28 (15–52)	28 (14–126)	0.43
ALT(U/l)	28 (8–81)	20.7 (5.8–114)	0.06
de-Ritis ratio	0.97 (0.57–2.88)	1.42 (0.72–3.59)	< 0.001
GGT (U/l)	45.7 (13.2–384)	64 (9-1578)	0.02
AP (U/l)	80 (32.9–667)	98.5 (40–756)	0.001
*Baseline PET parameters*			
SSTR-TV (cm^3^)	104.7 (2.9–1656)	156.2 (12.4–1610)	0.30
TL-SSTR (cm^3^)	892.3 (24.9-19148)	2357 (105.0-13194)	0.17
SUV_max_	36.5 (15.1-173.8)	50.2 (15.0-150.6)	0.19
SUV_peak_	25.3 (11.0-131.4)	31.1 (14.0-92.2)	0.11
SUV_mean_	9.8 (5.1–29.0)	11.9 (6.8–42.3)	0.15
Sites of metastases	N (%)	N (%)	
Bone	11 (30%)	21 (57%)	
Lymph node	24 (65%)	26 (70%)	
Liver	29 (78%)	32 (87%)	
Lung	1 (2.7%)	4 (11%)	
Visceral	13 (35%)	19 (51%)	
*Prior Treatments*	Prior i-PRRT N (%)	Between i-PRRT and re-PRRT N (%)	
Surgery	25 (68%)	3 (8%)	
Cold SSA	27 (73%)	35 (95%)	
Chemotherapy	3 (8%)	7 (19%)	
Locoregional procedures	11 (30%)	11 (30%)	
TKI	1 (3%)	1 (3%)	
RTX	2 (5%)	3 (8%)	
Immunotherapy/interferon	2 (5%)	1 (3%)	

Abbreviations: *CRP *C-reactive protein, *LDH *lactate dehydrogenase, *eGFR *estimated glomerular filtration rate, *AST *aspartate aminotransferase, *ALT *alanine aminotransferase, *de-Ritis ratio *AST/ALT ratio, *GGT *gamma-glutamyl transferase, *AP *alkaline phosphatase, *SSTR-TV *somatostatin receptor–positive tumor volume, *TL-SSTR *total lesion somatostatin receptor expression, *SUV *standardized uptake value, *SSA *somatostatin analogue, *TKI *tyrosine kinase inhibitor, *RTX *radiotherapy

The most common sites of metastases at initial PRRT included the liver (78%), lymph nodes (65%), visceral organs (35%), and bone (30%). The number of patients with metastases in these locations increased for re-PRRT. Prior to i-PRRT, most patients had undergone surgery (68%) and locoregional therapies (30%) as non-systemic treatments. As expected, a large proportion of patients received cold somatostatin analogues (SSAs, 73%). Further details can be found in Table 1.

### Treatment exposure and clinical course

Patients underwent a median of 4 (range 2–8) cycles of i-PRRT, with a median activity of 29.5 (range 13.3–60.8) GBq, and a median of 3 (range 1–4) cycles of re-PRRT, with a median activity of 22.2 (range 5.0–30.4) GBq.

### Safety and adverse events

**Fig. 3:**
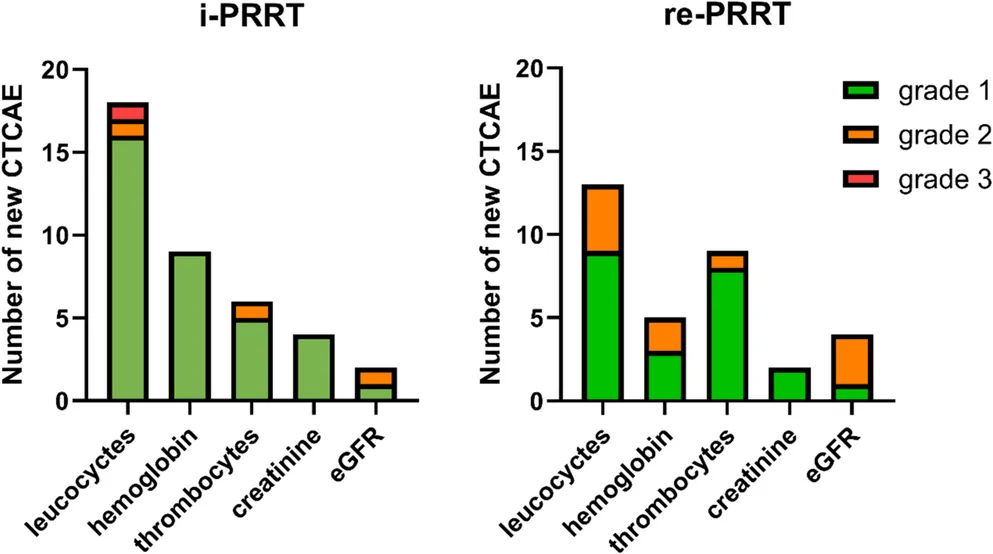
Classification of adverse events by severity according to CTCAE for i-PRRT and re-PRRT

Adverse events were predominantly low grade after re-PRRT (grade 1/2 toxicity for leucocytes, hemoglobin, thrombocytes, creatinine and eGFR in 35%, 14%, 24%, 5% and 11%, respectively), with no grade ≥ 3 events observed. After i-PRRT, however, one grade 3 leukopenia occurred (grade 1/2 toxicity for leucocytes, hemoglobin, thrombocytes, creatinine and eGFR in 46%, 24%, 16%, 11% and 5%, respectively; Fig. 3). An exploratory comparison of CTCAE-defined adverse events between patients treated with [¹⁷⁷Lu]Lu-DOTATOC and [¹⁷⁷Lu]Lu-DOTATATE revealed no significant differences in toxicity.

### Clinical course and differences in baseline characteristics between initial and repeat PRRT

The median interval between i-PRRT and re-PRRT was 36 months (range 15–104). During the interval, most patients received somatostatin analogues (35/37), while two did not due to treatment intolerance or non-adherence. Additional therapies included locoregional interventions (11/37), chemotherapy (7/37), surgery (3/37), radiotherapy (3/37), immunotherapy (1/37), and tyrosine kinase inhibitors (1/37). All patients experienced disease progression after i-PRRT despite intervening therapies. Compared to i-PRRT, median hemoglobin (13.2 vs. 12.2 g/dL; *p* < 0.01) and eGFR (85 vs. 74 mL/min/1.73 m²; *p* < 0.01) were lower at the beginning of re-PRRT, whereas GGT (45.7 vs. 64.0 U/L; *p* = 0.02), alkaline phosphatase (80.0 vs. 98.5 U/L; *p* < 0.01), and de-Ritis ratio (0.97 vs. 1.42; *p* < 0.001) were higher. No significant differences were found in the PET derived parameters like SSTR-TV (104.7 vs. 156.2 ml; *p* = 0.3), SUV_max_ (36.5 vs. 50.2; *p* = 0.1) and SUV_mean_ (9.8 vs. 11.9; *p* = 0.2) (Table 1).

### Treatment response and response dynamics across PRRT courses

**Fig. 4:**
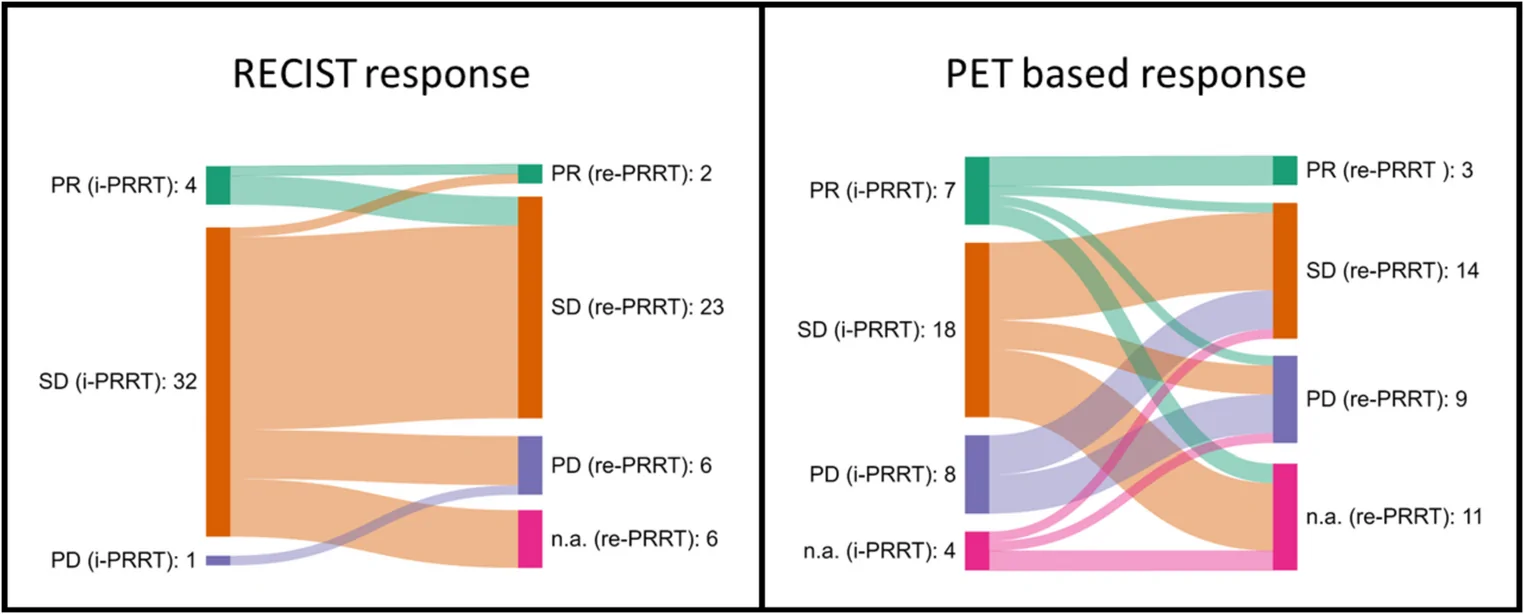
Sankeymatic diagrams showing response transitions between initial PRRT (i-PRRT) and retreatment (re-PRRT) based on RECIST (left) and PET-derived criteria (right). Flows indicate the number of patients shifting between partial response (PR), stable disease (SD), progressive disease (PD), and non-available (n.a.) categories. Non-available cases reflect patients without further assessable imaging follow-up

Applying RECIST criteria after the first two cycles of i-PRRT, 4/37 (11%) patients showed a PR (re-PRRT 2/31, 6%), while most patients remained SD (32/37, 86%; re-PRRT: 23/31, 74%). Applying PET response criteria after the first two cycles of i-PRRT, 7/33 (21%) patients showed a PR (re-PRRT 3/26, 11%), while 18/33 (55%) patients remained SD (re-PRRT 14/26, 54%) and 8/33 showed PD (24%; re-PRRT 9/26, 35%) (Fig. 4).

Response transitions between initial PRRT (i-PRRT) and retreatment (re-PRRT) are illustrated in Fig. 4. RECIST-based assessment indicated sustained morphologic disease control, with 23 of 32 patients remaining stable at re-PRRT and 5 exhibiting progression. Of the four patients achieving partial response after i-PRRT, two maintained a response while two transitioned to stable disease. PET-based evaluation revealed more heterogeneous response patterns: although stable disease was the most frequent category at re-PRRT (14 patients), progression occurred in 9 patients, and only 3 of 7 patients with an initial partial response preserved this status. PET response could not be assessed in 11 patients at re-PRRT, consistent with non-standardized imaging follow-up inherent to the retrospective study design. Consequently, response-transition analyses should be interpreted as exploratory.

**Fig. 5:**
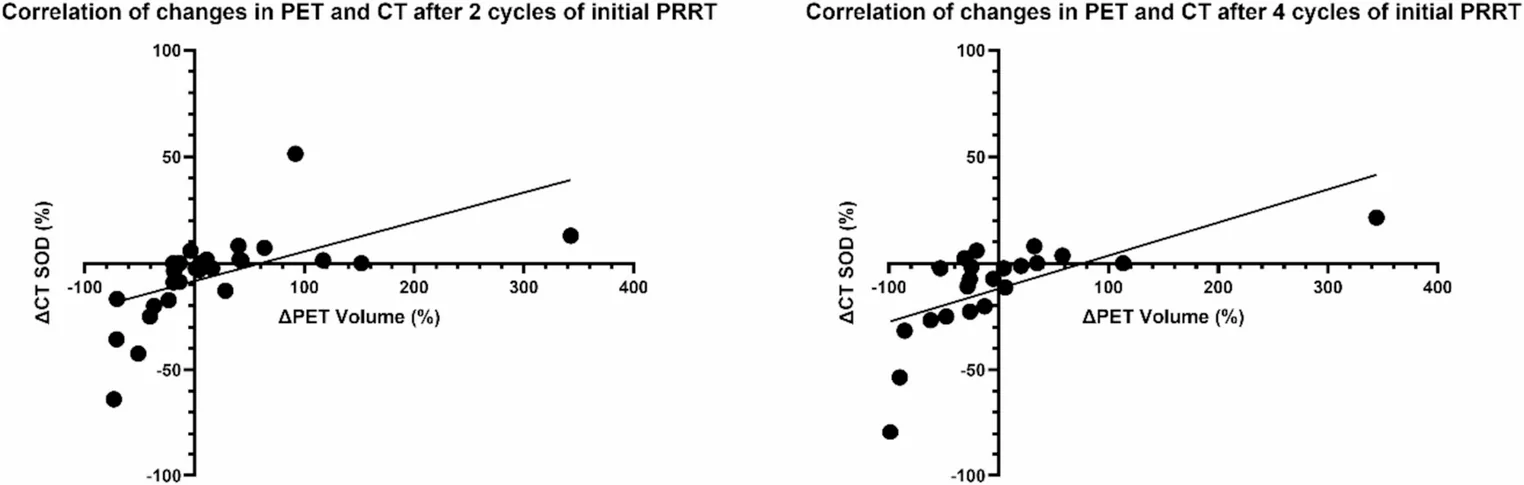
Correlation between the change in tumor volume on PET and the change in diameters on CT after 2 and 4 cycles of initial PRRT. The figure provides methodological context for the PET-based response analysis by illustrating the relationship between PET-derived tumor volume changes and RECIST-based morphologic changes. SOD = sum of diameter

Changes in SSTR-TV after i-PRRT strongly correlated with changes in the sum of diameters used for RECIST response assessment after two cycles (*r* = 0.83, *p* < 0.001), while a more moderate correlation was observed after four cycles (*r* = 0.64, *p* = 0.001; Fig. 5), supporting the internal consistency of PET-derived tumor burden assessment within this cohort.

### Progression-free survival and predictors of retreatment benefit

**Fig. 6:**
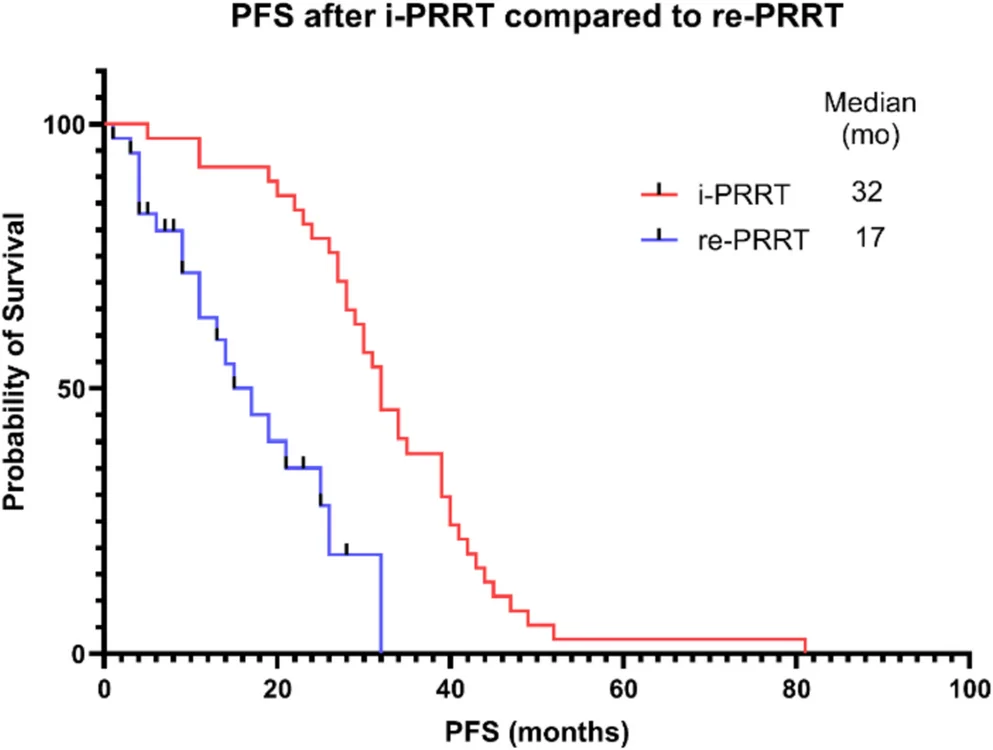
Kaplan-Meier curves of progression free survival after i-PRRT and re-PRRT

The median PFS1 after i-PRRT was 32 months and the median PFS2 after re-PRRT was 17 months (progression in 20/37 patients, Fig. 6).

In exploratory univariable Cox regression including baseline laboratory values and PET parameters, higher CRP was significantly associated with shorter PFS following i-PRRT (per mg/dl, HR 8.8, 95% CI 1.2–58.6; *p* < 0.05). This association was not observed for re-PRRT (per mg/dl, HR 0.79, 95% CI 0.13–3.8; *p* = 0.8), whereas higher LDH was significantly associated with shorter PFS after re-PRRT (per U/l, HR 1.007, 95% CI 1.000–1.014; *p* < 0.05). No baseline PET-derived parameter was associated with PFS.

**Fig. 7:**
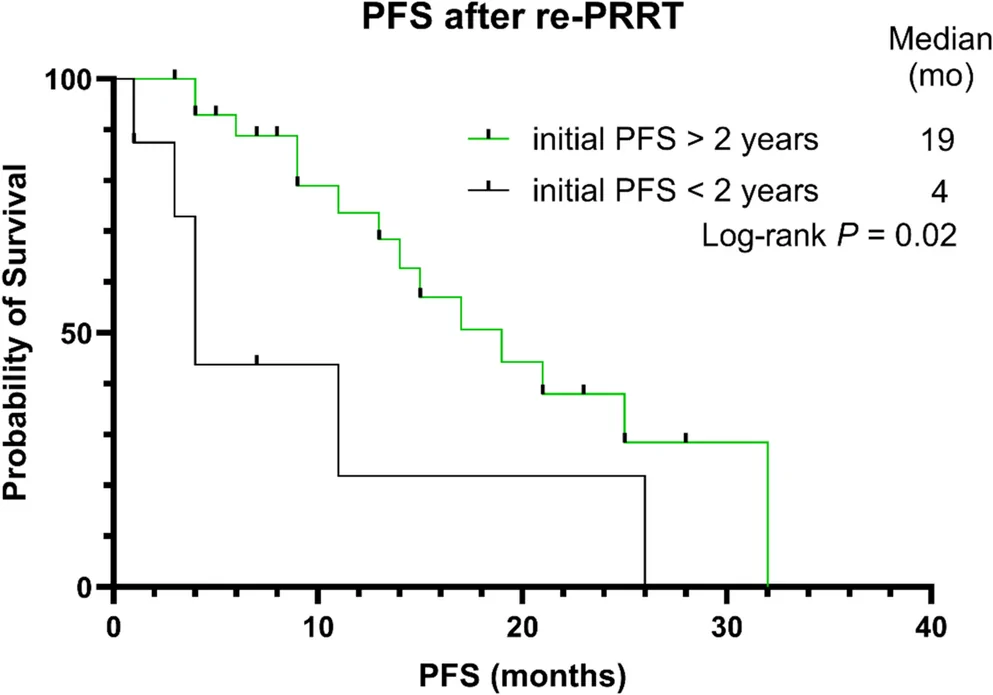
Kaplan–Meier curves of progression-free survival after re-PRRT, stratified by progression-free survival after i-PRRT (< 24 vs. ≥24 months)

Further Cox regression analysis demonstrated that longer PFS after i-PRRT was associated with prolonged PFS following re-PRRT (per month, HR 0.96, 95% CI 0.93–0.999; *p* < 0.05). As an exploratory analysis, patients were dichotomized using a 24-month PFS threshold after i-PRRT. Patients with longer initial disease control achieved a longer median PFS after re-PRRT (19 vs. 4 months; log-rank *p* = 0.02; Fig. 7). The 24-month cutoff was chosen based on the observed distribution of PFS after i-PRRT. Given the limited cohort size and small number of patients in the shorter-PFS group (*n* = 8), this cutoff should be interpreted as hypothesis-generating.

## Discussion

This retrospective analysis suggests that re-PRRT is a feasible and well-tolerated treatment option for selected patients with neuroendocrine tumors, demonstrating clinically meaningful anti-tumor activity despite shorter progression-free survival compared with initial therapy. The reduced PFS is consistent with the more advanced disease stage and prior treatment exposure typically observed in retreatment populations. Notably, our findings indicate that the duration of disease control following i-PRRT may serve as a pragmatic and clinically applicable marker to assist decision-making. The exploratory 24-month threshold may provide a pragmatic way to visualize this association, but should not be interpreted as a validated decision criterion. Rather, it generates a clinically plausible hypothesis that patients with more durable disease control after i-PRRT may be more likely to derive benefit from re-PRRT. In contrast, commonly assessed baseline laboratory and PET-derived parameters did not consistently predict outcomes across treatment settings.

Our findings are consistent with a growing body of evidence supporting the feasibility of re-PRRT in patients with neuroendocrine tumors, such as Rudisile et al., who evaluated re-PRRT in 35 NET patients and reported a PFS1 of 33 months and a PFS2 of 6 months [19]. The shorter PFS2 observed in their cohort may be attributable to greater population heterogeneity, including not only gastroenteropancreatic NET but also a higher proportion of lung NET, CUP NET, and paraganglioma, whereas our cohort predominantly comprised gastroenteropancreatic NET. Similar to our results, high-grade toxicities were rare, with only a single case of grade 3 hematotoxicity reported [19]. Another study described a PFS2 comparable to ours (14.6 months) in a mixed cohort of 181 patients with lung NET and gastroenteropancreatic NET. However, inclusion was limited to patients achieving a minimum PFS of 18 months after initial PRRT, likely resulting in a more favorable population than ours. Consistent with our observations, the authors reported very low rates of high-grade toxicity [20]. In a smaller cohort, Sitani et al. reported a PFS2 of 17 months following re-PRRT in NET patients who maintained disease stabilization for at least 12 months after initial PRRT, again without significant high-grade toxicities [21]. Variations in reported outcomes across studies may be explained by differences in patient selection criteria. Notably, the latter studies applied minimum response durations of 12 and 18 months after initial PRRT as inclusion criteria. Our study did not implement such strict thresholds, resulting in a cohort that may better reflect real-world clinical practice. Collectively, these data suggest that careful patient selection represents a critical factor when considering PRRT retreatment. In contrast to prior studies primarily reporting outcomes, our analysis specifically focuses on clinically applicable factors to guide patient selection for re-PRRT. Larger retrospective cohorts have similarly demonstrated shorter PFS following re-PRRT while highlighting substantial heterogeneity in outcomes, further underscoring the need for clinically applicable selection criteria.

A key finding of the present study is the association between the duration of disease control following i-PRRT and outcomes after re-PRRT. Patients with prolonged disease control after initial therapy experienced longer PFS following retreatment, suggesting that the duration of disease control may represent a readily available clinical parameter associated with retreatment outcome. However, this association may also reflect inherently more indolent tumor biology and the selection of fitter patients for re-PRRT, rather than a treatment-specific effect of retreatment itself. Given the retrospective design, prognostic and predictive effects cannot be disentangled. Consequently, the observed association should not be interpreted as a validated decision criterion but rather as a clinically accessible parameter that may complement established clinical factors when considering retreatment. The exploratory identification of a 24-month threshold provides a pragmatic illustration of this association but should not be interpreted as a validated cutoff. Instead, it generates the hypothesis that patients with more durable disease control after i-PRRT may be more likely to experience prolonged disease control following re-PRRT, a concept that requires prospective validation.

Importantly, retreatment decisions were not based on the early morphologic or exploratory PET response observed after two cycles of the initial PRRT course. Instead, re-PRRT was considered years later following renewed disease progression and was based on the overall clinical course, adequate SSTR expression on restaging PET/CT, preserved organ function, and multidisciplinary assessment. Consequently, even patients with limited early morphologic or exploratory PET response after i-PRRT could remain appropriate candidates for retreatment if they subsequently experienced a prolonged period of disease control.

Notably, in contrast to reports identifying SSTR-positive tumor volume as outcome-relevant [24], the selection process inherent to re-PRRT likely produced a cohort that differed substantially from unselected populations, potentially explaining the lack of statistical significance in our analysis. As baseline PET-derived parameters did not demonstrate consistent prognostic value, this finding highlights the continued relevance of clinical disease behavior over imaging metrics alone. While these findings require prospective validation, they support a more individualized approach to PRRT retreatment that incorporates the trajectory of prior therapeutic benefit.

The exploratory associations observed for CRP and LDH in our cohort may reflect distinct aspects of tumor biology and disease burden, but should be interpreted cautiously given the limited number of events and the absence of adjustment for multiple testing. CRP is widely recognized as a nonspecific marker of systemic inflammation and has been associated with adverse outcomes across multiple malignancies, likely representing a surrogate for tumor-related inflammatory processes [25–29]. In contrast, LDH is closely linked to tumor metabolism and cellular turnover and is frequently elevated in more advanced disease states [30–34]. The association between elevated LDH and shorter PFS following re-PRRT may therefore indicate a subgroup with more aggressive tumor behavior and diminished treatment responsiveness. Furthermore, patients receiving initial PRRT were generally fitter, whereas those undergoing re-PRRT exhibited slightly greater morbidity, reflected by lower hemoglobin and eGFR levels as well as elevated liver parameters suggestive of more advanced hepatic involvement. While these observations should be interpreted cautiously, they align with the broader concept that biochemical markers reflecting tumor dynamics may complement clinical parameters when refining risk stratification in the retreatment setting.

The median interval of 36 months between i-PRRT and re-PRRT and the limited use of additional systemic therapies likely reflects the generally indolent disease course characteristic of many well-differentiated NET compared to other tumors. However, patients considered for re-PRRT had progressed after initial therapy and frequently after additional intervening treatments. Notably, some individuals achieved meaningful responses to re-PRRT despite deriving limited benefit from the initial course. Most patients underwent further systemic or locoregional interventions between treatment lines, often with continued disease progression. Reduced effectiveness of systemic therapies in later lines is well recognized and is commonly attributed to tumor heterogeneity and treatment-driven clonal selection [35]. Nevertheless, re-PRRT continued to demonstrate clinically meaningful anti-tumor activity in this heavily pretreated population along with the favorable safety profile previously reported for PRRT, including in the NETTER-1 trial [9]. No grade ≥ 3 toxicities were observed during re-PRRT, which should be interpreted in the context of careful patient selection, the lower number of retreatment cycles, and systematic monitoring of laboratory and clinical parameters in routine practice. At the same time, patients undergoing re-PRRT exhibited significantly lower baseline hemoglobin levels and reduced renal function than before i-PRRT, reflecting a measurable cumulative treatment burden that had developed between treatment courses. These changes likely resulted from the combined effects of cumulative treatment exposure, disease progression, and additional systemic or locoregional interventions. Although they did not translate into clinically relevant high-grade toxicities during re-PRRT, they indicate that the absence of grade ≥ 3 adverse events should not be equated with the absence of cumulative treatment-related effects. Rather, these findings underscore the importance of careful longitudinal monitoring of hematologic and renal function when considering PRRT retreatment.

In our cohort, baseline and follow-up imaging were predominantly performed using DOTATOC PET/CT, allowing assessment of treatment response by both RECIST-based morphologic criteria and PET-derived metabolic parameters. For PET evaluation, we applied an exploratory volumetric classification based on ± 30% changes in SSTR-positive tumor volume. The ± 30% thresholds were selected pragmatically to facilitate within-cohort comparisons and were inspired by established response frameworks; however, they do not represent validated PET response criteria such as PERCIST, which is based on changes in SULpeak rather than tumor volume [36]. Furthermore, PET response assessment was unavailable in approximately one-third of patients undergoing re-PRRT, limiting the interpretation of response-transition analyses. Consequently, these findings should be considered exploratory and primarily hypothesis-generating. Nevertheless, PET-based assessments revealed response patterns that differed from RECIST classifications, under which most patients were categorized as having stable disease. This discrepancy likely reflects the greater sensitivity of PET, enabling three-dimensional quantification of total tumor burden, whereas RECIST primarily captures size changes in selected target lesions. These observations support further investigation into standardized PET-based response criteria and their potential role in clinical decision-making.

The present study has several limitations. First, the relatively small sample size and the retrospective design reflect real-world clinical decision-making, where retreatment is considered across heterogeneous patient populations and prior treatment exposures. Although the cohort size is limited, it is comparable to several previously published re-PRRT series. Second, the selection of patients undergoing re-PRRT introduces an inherent risk of selection bias, as this population may represent a clinically distinct subgroup. Additionally, therapies administered between initial and repeat PRRT were not standardized and may have influenced both SSTR expression and subsequent treatment responsiveness. Furthermore, progression-free survival may have been influenced by non-standardized imaging intervals inherent to retrospective clinical practice. Another potential source of confounding is the use of two different radiolabeled somatostatin analogues during the study period. Although exploratory analyses demonstrated no significant differences in CTCAE-defined toxicities between [¹⁷⁷Lu]Lu-DOTATOC and [¹⁷⁷Lu]Lu-DOTATATE, the study was not designed or sufficiently powered to compare radiopharmaceutical-specific efficacy. Consequently, residual confounding related to differences in peptide characteristics, cumulative administered activity, or radiopharmaceutical selection cannot be excluded. Although the cohort predominantly consisted of well-differentiated G1/G2 NET, a small number of patients with G3 or unknown tumor grade were included, reflecting routine clinical practice. The limited number of these cases precluded meaningful grade-specific analyses and is unlikely to have materially influenced the overall findings. Finally, overall survival was not evaluated because this retrospective cohort received heterogeneous subsequent therapies after re-PRRT, which would substantially confound survival analyses. Under these circumstances, progression-free survival was considered the more appropriate endpoint for assessing treatment-associated outcomes. Nevertheless, these findings add to the growing body of real-world evidence on PRRT retreatment and provide clinically relevant insight into patient selection. Prospective, preferably multicenter investigations are warranted to validate these observations and further refine retreatment strategies.

## Conclusion

In this real-world cohort, re-PRRT appeared to be a feasible and well-tolerated treatment option for selected patients with neuroendocrine tumors, demonstrating clinically meaningful activity despite shorter progression-free survival compared with initial therapy. The duration of disease control following i-PRRT emerged as a readily available clinical marker associated with outcomes after re-PRRT. An exploratory 24-month threshold may help visualize this relationship, but requires prospective validation before it can be used for treatment selection.

## Supplementary Information

Below is the link to the electronic supplementary material.

Supplementary Material 1

## Data Availability

The main data presented in this study are available in the article. Detailed information about the image analysis or the progression free survivals of the subjects presented in this study are available on reasonable request from the corresponding author.
