# Supplementary material for: Patient selection for PRRT rechallenge in neuroendocrine tumors: impact of initial response duration on outcomes
Source: Eur J Nucl Med Mol Imaging. 2026 Aug 14;53(13):8322–32. doi: 10.1007/s00259-026-08130-8 (PMC13727927; doi:10.1007/s00259-026-08130-8)
Supplement: Supplementary file 1 [file 259_2026_8130_MOESM1_ESM.docx]

**Radiochemistry**

*Synthesis of [^68^Ga]Ga-DOTATOC*

Radiochemical synthesis of [⁶⁸Ga]Ga-DOTATOC was performed using a fully automated, GMP-compliant procedure on a GRP® module (SCINTOMICS GmbH, Fürstenfeldbruck, Germany) connected to a ⁶⁸Ge/⁶⁸Ga generator (Eckert & Ziegler, Berlin, Germany) and equipped with a disposable single-use cassette kit (ABX, Radeberg, Germany). The generator eluate ([⁶⁸Ga]GaCl₃ in 0.1 M HCl) was transferred to a cation-exchange cartridge, eluted with 5 N NaCl, and added to a solution containing 20 μg DOTA-TOC (ABX, Radeberg, Germany) in HEPES buffer. The reaction mixture was heated at 125 °C for 6 minutes. The labeled product was subsequently trapped on a Sep-Pak C18 cartridge, washed with water, and eluted with an ethanol/water mixture (50/50, v/v). The eluate was passed through a sterile 0.22-μm filter into a sterile vial and diluted with phosphate buffer solution to a final volume of 15 mL. Radiochemical purity was assessed by gradient high-performance liquid chromatography (HPLC) and thin-layer chromatography. Additional quality control included determination of ethanol content, pH, radionuclide purity, sterility, and endotoxin levels.

*Synthesis of [^177^Lu]Lu-DOTATOC*

Radiolabeling of [¹⁷⁷Lu]Lu-DOTATOC for therapeutic administration was performed by adding 8 GBq [¹⁷⁷Lu]LuCl₃ (ITG GmbH, Garching, Germany) in 200 μL of 0.04 M HCl to a reaction mixture containing 200 μg DOTA-TOC (ABX, Radeberg, Germany) and 7 mg gentisic acid in 525 μL of 0.4 M sodium acetate buffer (pH 5.2). The reaction mixture was heated at 100 °C for 35 minutes. After cooling, the solution was handled under sterile conditions, diluted with 14 mL saline, and passed through a sterile 0.22-μm filter into a sterile 20-mL syringe. Each batch of [¹⁷⁷Lu]Lu-DOTATOC underwent quality control prior to release, including assessment of radiochemical purity by gradient high-performance liquid chromatography (HPLC) and thin-layer chromatography. Additional testing included determination of pH, endotoxin levels, and filter integrity using a bubble point test.

**Imaging Protocol**

PET/CT scans were performed using either a Siemens Biograph mCT 64 or a Siemens Biograph mCT 128 Flow scanner (Siemens Healthineers, Knoxville, TN, USA). Patients were instructed to void prior to imaging. All PET acquisitions were obtained in three-dimensional mode. Images were reconstructed iteratively using an ordered-subset expectation maximization algorithm (OSEM). For the Biograph mCT 64, reconstruction employed 3 iterations and 24 subsets followed by a 2-mm full-width at half-maximum (FWHM) Gaussian filter (TrueX). For the Biograph mCT 128 Flow, reconstruction used 3 iterations and 21 subsets with a 2-mm FWHM Gaussian filter (TrueX+TOF UltraHD). The reconstructed spatial resolution ranged from approximately 4.7 mm (Biograph mCT 128 Flow) to 5.7 mm (Biograph mCT 64) FWHM.

Table 1: Number of patients with PET or CT at restaging timepoints

|  | i-PRRT |  | re-PRRT |  |
| --- | --- | --- | --- | --- |
|  | After 2 cycles | After 4 cycles | After 2 cycles | After 4 cycles |
| CT | 37 | 31 | 31 | 18 |
| PET | 33 | 25 | 26 | 15 |
